# Unmet needs and perspectives in rheumatoid arthritis-associated interstitial lung disease: A critical review

**DOI:** 10.3389/fmed.2023.1129939

**Published:** 2023-03-16

**Authors:** Anna Stainer, Antonio Tonutti, Maria De Santis, Francesco Amati, Angela Ceribelli, Gabriele Bongiovanni, Chiara Torrisi, Antonio Iacopino, Giuseppe Mangiameli, Stefano Aliberti, Carlo Selmi

**Affiliations:** ^1^Department of Biomedical Sciences, Humanitas University, Milan, Italy; ^2^Division of Respiratory Medicine, IRCCS Humanitas Research Hospital, Milan, Italy; ^3^IRCCS Humanitas Research Hospital, Milan, Italy; ^4^Division of Rheumatology and Clinical Immunology, IRCCS Humanitas Research Hospital, Milan, Italy; ^5^Department of Radiology, IRCCS Humanitas Research Hospital, Milan, Italy; ^6^Division of Thoracic Surgery, IRCCS Humanitas Research Hospital, Milan, Italy

**Keywords:** progressive pulmonary fibrosis, biomarkers, immunology, precision medicine, rheumatoid arthritis, interstitial lung disease, clinical trials, lung ultrasonography

## Abstract

Rheumatoid arthritis (RA) is a chronic systemic autoimmune disease characterized by synovitis as the most common clinical manifestation, and interstitial lung disease (RA-ILD) represents one of the most common and potentially severe extra-articular features. Our current understanding of the mechanisms and predictors of RA-ILD is limited despite the demonstration that an early identification of progressive fibrosing forms is crucial to provide timely treatment with antifibrotic therapies. While high resolution computed tomography is the gold standard technique for the diagnosis and follow-up of RA-ILD, it has been hypothesized that serum biomarkers (including novel and rare autoantibodies), new imaging techniques such as ultrasound of the lung, or the application of innovative radiologic algorithms may help towards predicting and detecting early forms of diseases. Further, while new treatments are becoming available for idiopathic and connective tissue disease-associated forms of lung fibrosis, the treatment of RA-ILD remains anecdotal and largely unexplored. We are convinced that a better understanding of the mechanisms connecting RA with ILD in a subgroup of patients as well as the creation of adequate diagnostic pathways will be mandatory steps for a more effective management of this clinically challenging entity.

## Introduction

Rheumatoid arthritis (RA) is a chronic inflammatory disease in which an autoimmune mechanism causes chronic inflammation which predominantly involves the synovia at the peripheral joints (1). Although the disease etiology remains largely unknown, genetic predisposition, environmental triggers, and aberrant immune system activation are well established factors determining RA pathogenesis (2). A large proportion of patients report extra-articular manifestations, including cardiovascular, respiratory, and cutaneous involvement (3). The presence of extra-articular manifestations may in some cases predate the clinical onset of arthritis, may require specific management measures, and ultimately impact therapy (3, 4). Focusing on the respiratory manifestations of RA, it has been estimated that lung disease accounts for 10%–20% of mortality in subjects with RA, being inferior only to cardiovascular events (5). While the lung parenchyma, airways, pleura, and vasculature may all be affected, RA-associated interstitial lung disease (RA-ILD) is the most common and potentially severe manifestation, as it can present with a progressive fibrosing phenotype (6). Acute exacerbations of RA-ILD are defined as a rapidly progressing, potentially life-threatening respiratory decline characterized by new extensive alveolar abnormalities superimposed on underlying pulmonary fibrosis (7). Acute exacerbations are a rare but severe complication carrying a 12% to 64% mortality (8–11). To provide a better overview of RA-ILD, we will herein review the prevalence, risk factors, clinical characteristics, and therapeutic perspective of RA-ILD.

### Prevalence, incidence, and mortality of RA-ILD

It has been estimated that RA-ILD explains about 8% of all cases of ILD (12). The prevalence of IL among patients with RA ranges between 1.8% and 67% and according to a recently published meta-analysis, the prevalence of clinically detected RA-ILD is also lower than radiologically detected cases (13). Chest high-resolution computed tomography (HRCT) is the most sensitive technique to screen for the presence of ILD in patients with RA and allows its characterization and quantification (14). The presence of symptoms and signs (i.e.: exercise dyspnea, cyanosis, inspiratory velcro-like crackles, digital clubbing) makes “clinically-driven” detection of RA-ILD ineffective and leads to delayed diagnosis at later stages (15). Thus, the use of different case finding methods explains, at least in part, the heterogeneity of RA-ILD prevalence that is reported in the literature.

Second, with the adoption of HRCT in clinical practice, an increase in RA-ILD prevalence has been observed over time (16). ILD has been detected in up to 7.5% subjects with early RA (17), while interstitial lung abnormalities (ILA, *vide infra*) may be more common (18). It has been estimated that 10% patients with established RA have clinically significant ILD (i.e., signs and symptoms, latent respiratory insufficiency, severe lung function impairment) (19). Moreover, ILD can precede RA clinical onset in a significant proportion of cases (20). Third and last, genetic susceptibility can be hypothesized to explain geographical differences (21).

While RA-associated general mortality has decreased over the last decades, mortality due to RA-ILD remained stable (15, 19) resulting in a 3–10 times higher risk of death in patients with RA-ILD compared to patients with RA without lung involvement (14, 20). RA-ILD not only increases the risk of all-cause and respiratory mortality, but also seems to be associated with elevated risk of cancer-related mortality (22) with pulmonary malignancy being the most common cancer-related cause of death in patients with RA, especially if ILD is present (22–24). The incidence of lung cancer is higher in patients <60 years with rheumatic disease-associated ILD (RD-ILD) than patients without rheumatic disease (25) and the incidence of lung cancer in RA-ILD is comparable to idiopathic pulmonary fibrosis (IPF) (26) but how these data apply to RA is unclear. Last, a trend towards a mortality reduction is associated with the early diagnosis of RA-ILD at HRCT and with the use of immunosuppressive therapy (27), while it has been demonstrated that diagnostic delay in RA-ILD diagnosis leads to increased mortality (28).

### Risk factors and prognostic factors of RA-ILD

Established risk factors for RA-ILD are summarized in Table 1 and include demographics such as older age, male sex, obesity, and smoking history, along with the presence of respiratory comorbidities (22, 29). In addition, RA disease features, such as longer disease duration (13), high disease activity (22) and serum autoantibodies, in particular rheumatoid factor (RF) and anti-citrullinated protein antibodies (ACPA) significantly increase the risk of developing ILD. Furthermore, novel emerging biomarkers seem to play a prognostic role (27, 29) while genetic risk factors such as gain-of-function MUC5B promoter variant rs35705950 increase the risk of ILD in patients with RA and are associated with the usual interstitial pneumonia (UIP) pattern at HRCT (30) apart from being associated with IPF (31). Additionally, single-nucleotide polymorphisms (SNPs) in TERT, TOLLIP, and FAM13A loci have also been associated with pulmonary fibrosis in patients with RA (32).

**Table 1:** Established risk factors for ILD occurrence in patients with RA.

**Demographics**
Older age
Male sex
Smoking history
**Comorbidities**
Obesity
Previous respiratory disease
**RA-associated features**
Long disease duration
High disease activity
Seropositivity for RF and/or ACPA
**Genetics**
MUC5B promoter variant rs35705950

While time-dependent decline in lung function correlates with mortality in RA-ILD (13, 33), other prognostic factors include older age, male sex, smoking habit, or the presence of comorbidities (13, 34) such as RA disease activity and the use of systemic glucocorticoids (13, 34). Additional poor prognostic features include pleural effusion, short time between RA diagnosis and ILD occurrence (35), as well as the radiologic pattern at HRCT with UIP pattern predicting mortality (13, 33, 36) and correlating with an increased risk of acute exacerbations and lung cancer (36).

### Progressive pulmonary fibrosis and RA-ILD

According to the Fleischner Society glossary, fibrosing ILD is defined in the presence of reticular abnormalities, traction bronchiectasis and bronchiolectasis, architectural distortion, and/or honeycombing on HRCT scan (37–39). These radiologic changes reflect the exuberant deposition of extracellular matrix within the pulmonary interstitium, and may lead to the development of progressive fibrosing ILD in a subset of patients (39).

Progressive pulmonary fibrosis (PPF) has been defined as the progression of at least two domains among clinical and/or functional and/or radiological status, occurring within 1 year, without any alternative explanation, in a patient with an established diagnosis of fibrosing ILD other than IPF (40). One third of patients with RA-ILD develop PPF (12, 41, 42), particularly when a UIP pattern is described at HRCT, although a minority of subjects with these features do not progress (43). PPF is associated with increased mortality and unfavorable outcomes (44) and the antifibrotic drug nintedanib is now recommended in this subset of patients (40), thus making an early diagnosis a major clinical need.

### Comorbidities in RA-ILD

Several comorbidities can impact the course of RA-ILD, affecting disease control and leading to impaired quality of life. According to Mena-Vázquez et al. ILD is independently associated with multimorbidity in patients with RA, with the most frequent comorbid conditions being traditional cardiovascular risk factors, depression, and osteoporosis (45).

Among respiratory comorbidities, RA-ILD can be associated with airway disease, including COPD, bronchiectasis and asthma (46). RA-ILD patients with COPD or emphysema have higher mortality risk in different cohorts (47–49). Interestingly, pre-existing COPD has been associated with a higher incidence of ILD in newly diagnosed RA patients (50). Bronchiectasis in RA are associated with increased risk of infections *per se* (46) and in patients treated with biologic disease modifying anti-rheumatic drugs (DMARDs) (51). RA-ILD is a risk factor for pneumonia (51, 52), in particular when associated to an organizing pneumonia pattern, and with daily doses of prednisone exceeding 10 mg. (52) A relevant concern in terms of infections is also represented by COVID-19 (53), since patients with RA are at increased risk of developing severe COVID-19, the risk appearing even higher in those with pre-existing ILD (54, 55).

Sleep disorders are frequently associated with RA. A recent meta-analysis has shown that the incidence of obstructive sleep apnea syndrome (OSAS) is 29.8% among RA cohorts, however with a significant heterogeneity, and high BMI is the principal risk factor (56). Although the epidemiology of sleep disorders is still blurred in RA-ILD, it is reasonable to consider OSAS as a significant complication in this subgroup of patients due to its relevance in other ILDs, including IPF, and can cause an extremely poor sleep quality that correlates with poor quality of life (57, 58). Thus, OSAS and prolonged oxygen desaturation during sleep have been associated with a worse prognosis in IPF, both in terms of mortality and clinical progression. (59)

Pulmonary hypertension associated to interstitial lung disease is an established clinical entity, owing to class III World Health Organization (WHO) classification (60). However, pulmonary arterial hypertension (PAH, i.e., class I WHO) can represent a rare complication patients suffering from RA (60, 61). Also, the prothrombotic effect associated to chronic inflammation increases the risk of venous thromboembolism (62), and chronic thromboembolic pulmonary hypertension should be taken into account when approaching to the differential diagnosis of pulmonary hypertension in patients with RA and RA-ILD. Interestingly, the dominating cause of pulmonary hypertension may change over time, making the diagnostic and therapeutic process more challenging (63).

## Traditional and novel biomarkers in RA-ILD

Biomarkers are measurable indicators of biologic and pathologic processes (64), and include well-established serum autoantibodies used in routine clinical or research settings in addition to RF and ACPA and non-autoimmune markers of lung damage. Table 2 summarizes the main established and investigational biomarkers in RA-ILD.

**Table 2:** Established and candidate biomarkers for RA-ILD; the proposed mechanistic links and setting of use are specified for all markers.

Biomarker	Pathogenic and/or clinical association(s)	Availability
Autoantibodies		
Rheumatoid factor (65–73)	Incidence and progression of ILD in RA correlate with RF titers; fibrosing ILD and UIP (especially IgA-RF)	+ (+/− isotypes)
ACPA (74–81)	Incidence and severity of ILD in RA correlate with ACPA titers; fibrosing ILD, UIP (especially ACPA repertoire expansion), NSIP (secretory ACPA)	+ (Repertoire for research purposes)
anti-CEP-1 (82)	Positive association with ILD in non-smokers	Research
anti-citrullinated Hsp90 (83)	Positive association with ILD	Research
Anti-PAD2 (84)	Negative association with ILD	Research
Anti-PAD3/4XR (85)	Positive association with ILD in non-smokers	Research
Anti-CarP (86)	Positive association with ILD at increasing titers	Research
Anti-MAA (87)	Positive association with ILD (IgA and IgM)	Research
Anti-class I (88)	Negative association with ILD	Research
Anti-MICA (88)	Positive association with ILD	Research
Antisynthetase (89–91)	Positive association with ILD, especially NSIP; ASSD overlap vs RA misdiagnosis	+
*Cytokines*		
IL-4 (92)	Increased in patients with RA-ILD	Research
IL-11 (93, 94)	Correlates with ILD severity and disease activity	Research
IL-13 (95)	Correlates with the extent of fibrosis	Research
IL-18 (96)	Increased in patients with RA-ILD	Research
IL-33 (93, 94)	Correlates with ILD severity	Research
Circulating factors with immunologic implication		
KL-6 (97–100)	Correlates with lung damage	+

### Rheumatoid factors (RF)

RF is represented by immunoglobulins, mainly IgM but also IgG and IgA, directed towards the constant (Fc) portion of another immunoglobulin (101); serum RF is positive in up to 80% of patients with RA, albeit with lower specificity (101) since non-rheumatic conditions (e.g., infective endocarditis, hepatitis B and C, primary biliary cholangitis, lymphoma) and rheumatic diseases other than RA (e.g., Sjogren’s syndrome, cryoglobulinemia) manifest different degrees of RF positivity (102). RF can be tested with different laboratory methods, including latex fixation test, Waaler-Rose reaction, and enzyme linked immunosorbent assay (ELISA) (103), with the latex fixation test and Waaler-Rose reaction capable of detecting only IgM-RF, while immunoassays can also identify IgG and IgA isotypes, possibly increasing the diagnostic sensitivity (104, 105).

RF positivity is an established risk factor for ILD development in patients with RA (65–67). It has been demonstrated that the prevalence and incidence of RA-ILD correlates with serum titers of RF (68) and high-titer RF is associated with an increased risk of progression and elevated mortality in patients with RA-ILD (69), along with a more aggressive form of RA with a higher risk of erosions (70). Moreover, signs of advanced fibrosis at HRCT (including honeycombing) have been associated with RF seropositivity in patients with RA-ILD (69).

In contrast to IgG and IgM RF isotypes, IgA-RF correlates with more severe articular disease, development of bone erosions, and increased prevalence of extra-articular manifestations including ILD (71, 72) with a higher prevalence of UIP pattern at HRCT (73). Despite this preliminary evidence, the clinical significance of different RF isotypes needs to be further explored in longitudinal studies.

### Anti-citrullinated protein antibodies

Serum ACPA are associated with an increased risk of ILD in patients with RA (74) in a titer-dependent manner (75). Also, the prevalence of ILD at disease onset is higher among subjects with high ACPA titers (76). There is also a direct correlation between ACPA and disease severity in terms of clinical presentation (i.e., symptoms, signs, presence of respiratory insufficiency), worse lung function, and extent of ILD on HRCT (77). In a meta-analysis by Zhu and colleagues, ACPA status predicted ILD in RA and was significantly associated with an increased risk of fibrosing ILD (78) at degrees correlating with ACPA titers (77). Since signs of fibrosis at HRCT are an established risk factor for PPF (40), patients with RA testing positive for ACPA could be at higher risk for PPF development and may warrant a closer monitoring of lung changes.

ACPA formation against different citrullinated peptides and epitope spreading, which is the development of immunity against self-antigens release during autoimmune responses (79), are established mechanisms in the pathogenesis of RA (80). As such, as RA progresses, the ACPA repertoire (i.e., the number of different autoantigens and specific moieties recognized by ACPA) expands. This phenomenon has been associated with an increased risk of fibrosing ILD, lower lung volumes and DLCO, and higher prevalence of UIP pattern at HRCT (81). This is relevant considering that such functional and radiological features can predict PPF and suggests that analyzing the ACPA repertoire during the disease course may help individuating patients with RA-ILD at high risk of PPF.

Among autoantibody subtypes, patients testing positive for serum ACPA with secretory components have more frequently a nonspecific interstitial pneumonia (NSIP) pattern at HRCT, in contrast to what is commonly seen in RA-ILD (73).

### Novel autoantibodies in RA-ILD

Among the non-classical autoantibodies putatively correlated with ILD in patients with RA, anti-citrullinated alpha-enolase peptide 1 (anti-CEP-1) have been identified as a subset of ACPA associated with erosive RA and ILD (82), particularly at high titers (106), and have been proposed for early detection of RA-ILD in at risk non-smoking patients (82). Anti-citrullinated heat shock protein 90 (Hsp90) antibodies are found in a subset of patients with RA-ILD, but not in patients with RA without ILD or in other forms of ILD (83). They are also present in the bronchoalveolar lavage fluid (BALF) (107) while autoreactive Th1 lymphocytes directed towards citrullinated Hsp90 have been detected in the peripheral blood of patients with RA-ILD (108). Peptidylarginine deaminase (PAD) is the most important enzyme causing citrullination (109), as demonstrated for the oral bacterium *P. gingivalis* (110). Autoantibodies against the human PAD isoforms PAD2, PAD3 and PAD4 have been detected in patients with RA (109) and may be useful in the risk stratification for lung disease. Anti-PAD2 antibodies have been described in a subset of patients with RA characterized by milder articular damage, as well as less frequent and less severe extra-articular manifestations, especially ILD (84), whereas anti-PAD4 antibodies correlate with a more aggressive disease (111). A subgroup of patients with RA possesses cross-reactive antibodies towards both PAD3 and PAD4, named anti-PAD3/4XR antibodies, that may predict ILD occurrence, especially in never-smoking patients (85), an association not found for anti-PAD3 or anti-PAD4 antibodies alone. Recent data demonstrate a possible association between double anti-PAD3 and PAD-4 positivity with both ILD and more erosive disease and the authors have hypothesized that such patients might have anti-PAD3/4XR positivity (112). Serum anti-carbamylated proteins antibodies (anti-CarP) have been reported at higher frequency in patients with RA-ILD compared to RA patients without ILD, independent of the smoking status (86). Malondialdehyde-acetaldehyde adducts (MAA) are highly expressed in the lung tissue from patients with RA-ILD, and antibodies against MAA (anti-MAA) have been associated with RA-ILD (87), especially high titers and the IgA or IgM isotypes (87). Furukawa and Colleagues have described the presence of autoantibodies to human leukocyte antigen (HLA) class I (anti-class I) and HLA class I related chain A (anti-MICA) in a cohort of patients with RA. Notably, higher levels of anti-MICA antibodies and higher values of the anti-MICA/anti-class I ratio were found in patients with RA-ILD, compared to patients without lung involvement (88). Antisynthetase antibodies are directed towards aminoacyl-tRNA-synthetase complex and are mainly found in a cluster of patients with inflammatory myositis, namely the antisynthetase syndrome (ASSD) (113) with NSIP as the most common ILD pattern observed at HRCT (114). In a cohort of patients with RA, the prevalence of serum antisynthetase antibodies was 6%, and ILD occurred more frequently (57%) in seropositive than seronegative (22%) patients. Specifically, anti-PL-7 was the most frequently reported among antisynthetase antibodies, whereas a low prevalence of anti-Jo-1 was observed; this contrasts with what is commonly seen in ASSD cohorts, where anti-Jo-1 is the most common antibody. Furthermore, opposite to RA-ILD with conventional antibodies like RF and ACPA, NSIP was the most frequent pattern at HRCT in antisynthetase antibody-positive subjects with RA (89). The association of antisynthetase antibodies and RA-ILD was confirmed in an independent cohort (90), and a case of anti-EJ and ACPA positive RA with ILD-only onset was also reported (115). Remarkably, ASSD is frequently misdiagnosed and treated as RA, especially when arthritis is the predominant manifestation (91).

### Routine laboratory tests

Among tests usually performed in the clinical setting, an unsuspected role has been proposed for serum uric acid, for which higher levels were observed in RA-ILD with a prevailing UIP pattern at HRCT (116). Serum uric acid is already included in the DETECT algorithm for early detection of pulmonary arterial hypertension in patients with systemic sclerosis (SSc) (117). Higher neutrophil and monocyte counts are independent predictors of mortality in RA-ILD, particularly when both are elevated (118).

### Other serum biomarkers

Among cytokines, serum titers of IL-4 (92) and IL-18 (96) are increased in patients with ILD, compared to the general RA population while serum IL-13 is increased in patients with RA-ILD and correlates with the extent of fibrosis at HRCT (95). However, such observations warrant further investigation since, as an example, IL-4 is a strict autocrine cytokine and serum levels may not differ even between subjects with IL-4-dependent diseases and healthy controls (119). Both arthritis and ILD severity correlate with the presence and serum concentrations of IL-11 and IL-33, independent of the RF and ACPA status, with the former being also associated with RA disease activity (93, 94). Within the IL23-IL17 axis, Zhang and colleagues reported that lung fibroblasts from patients with RA-ILD express significantly higher levels of the IL-17A receptor (IL-17RA) compared to patients without ILD or IPF (120) while IL-23 contributes to the epithelial-mesenchymal transition in the lung of RA-ILD (121). While the role of IL-17 and IL-23 remains elusive, these results support the use of monoclonal antibodies against IL-17 (e.g., secukinumab, ixekizumab) and IL-23 (e.g., guselkumab, risankizumab, and ustekinumab) which are currently used in spondyloarthritis, psoriasis and psoriatic arthritis, and inflammatory bowel disease for RA-ILD despite being proven ineffective on the articular manifestations of RA (122, 123). Krebs von den Lungen 6 (KL-6, a glycoprotein expressed by type II alveolar cells) serum levels correlate with lung damage in patients with ILD (97) with higher baseline values associated with mortality in RA-ILD, especially with a UIP pattern at HRCT (98). Changes in KL-6 values over time may predict acute exacerbations of RA-ILD (99) to make routine tests a putative screening method for ILD in patients with RA (100). In combination with KL-6, the oncological markers CA 19-9, CA 125, and CEA correlate with the presence and severity of ILD in patients with RA (124) while serum HE4, a biomarker for ovarian cancer, may identify RA cases at risk for subclinical ILD (125). It was demonstrated that serum onco-marker CA 15–3 is a valid alternative to KL-6, with comparable sensitivity and specificity in differentiating fibrosing and non-fibrosing ILD (126). Other proposed molecules involved at different levels in the immune, inflammatory, and fibrotic response characterizing RA-ILD include matrix metalloproteinase 7 (MMP-7), C-X-C motif chemokine ligand 10 (CXCL10) (127), Dickkopf 1 (DKK1) (128), and soluble programmed death 1 (sPD-1) (129). Circulating endothelial progenitor cells (EPCs) are associated with the repair of alveolar damage and are increased in RA-ILD compared to RA patients without lung involvement. However, their levels are lower in comparison to patients with IPF (130). While we acknowledge that observed differences refer to tests performed only for research purposes (67, 131), it should also be noted that non-coding RNAs (132, 133) and metabolomic profiling (134) have also been proposed with promising preliminary results.

### Biomarkers in RA-ILD: Unmet needs and research questions

Except for traditional RA autoantibodies (i.e., RF and ACPA), no biomarker is currently used in clinical practice for the screening of RA-ILD, thus, further studies are required. First, it is of critical importance to individuate at baseline (or as early as possible) which patients with RA are at high risk of developing ILD. Second, once RA-ILD is established, there is a need to understand which subjects are likely to develop clinically significant disease or are going to require specific therapies (even in the presence of subclinical disease). Third, since fibrosis and PPF are major concerns in the management of RA-ILD, biomarkers are required for early identification of patients at risk of developing progressive fibrosing ILD. Fourth, there is an urgent need to understand whether antifibrotic therapy can be started only when PPF has established or, vice versa, whether there is any benefit from starting such therapy in patients ‘at high risk of PPF’. Fifth, since ‘ILD’ does not always mean ‘fibrosis’, biomarkers could help in discriminating different ‘treatable traits’ when clinical worsening occurs (e.g., progressive fibrosis versus inflammation versus superimposed infection, etc.). Sixth, predictive biomarkers that inform us of therapeutic effects are warranted.

## Imaging in RA-ILD

To date, there are neither consensus statements or guidelines / recommendations on radiologic screening and follow-up of pulmonary involvement in RA, despite HRCT remaining the preferred tool for the identification of lung involvement in RA with a better sensitivity compared to chest X ray at early stages (135). Of importance, HRCT allows to discriminate between inflammatory and fibrotic lesions (136) with prognostic implications (33).

### Preclinical thoracic findings

Lung involvement may predate the onset of RA, particularly with ancillary signs suggesting rheumatic involvement including RA-ILD, pleural effusion, pleuritis, bronchiectasis, rheumatoid nodules, pulmonary vascular diseases, and drug-associated lung complications (137).

ILAs, incidental findings involving at least 5% of lung parenchyma at HRCT in individuals in which ILD in not suspected (38) can be the first detectable sign both in patients with early and longstanding RA (138), with the latter having more frequent HRCT abnormalities (139). Gabbay et al. (140) detected ILAs in 44% of RA cases screened for lung involvement while others found HRCT abnormalities in nearly 50% of the patients with no respiratory symptoms. Factors significantly associated with HRCT abnormalities were age older than 40 years, positive tests for IgM-RF, hypoxia at rest, and lung function test evidence of distal airway disease (141). A lower incidence (22%) has been reported in a retrospective study of 293 patients with RA undergoing HRCT; 29% of these manifested progression over 4.4 years, particularly with subpleural distribution and higher baseline ILA extent. HRCT scans were performed for non-pulmonary indications in 46% patients, and ILAs were detected in a considerable proportion (44%) of these subjects. This supports the hypothesis that pulmonary involvement in RA is largely underdiagnosed (142). A 57% progression rate in ILAs has been described in a different study, largely related to past cigarette smoking (143).

### ILD patterns at imaging

In patients with RA, UIP is the most common pattern at presentation, followed by NSIP while other types of ILD, i.e., organizing pneumonia (OP), desquamative interstitial pneumonia (DIP) and lymphocytic interstitial pneumonia (LIP), are found less frequently (144, 145). Typical UIP pattern is characterized by heterogenous honeycombing of the pulmonary bases and periphery, peripheral basilar predominant reticular abnormalities, and architectural distortions. However, the presence of anterior upper lobe honeycombing sign, where honeycombing is distributed both in the anterior upper lobes as well as in pulmonary bases, or the presence of exuberant honeycombing sign, where honeycombing is hypertrophic and distributed across multiple layers, are frequently associated with RD-ILDs, including RA-ILD (146, 147). The UIP pattern has been associated with an increased mortality in RA-ILD in different studies (137, 148–150) while Yunt et al. did not report any difference in survival between subjects with definite UIP versus those with possible UIP (137). A recent meta-analysis confirmed that UIP pattern at HRCT, presence of emphysema, and both the occurrence and number of acute exacerbations were associated with increased mortality in RA-ILD (151). Different from UIP, the NSIP pattern is characterized by ground-glass opacities (GGO), fine reticulation or traction bronchiectasis within GGO, and airspace consolidation while honeycombing is rarely present (152). Patients with NSIP develop pulmonary involvement at younger age and longer after RA diagnosis compared to the UIP pattern (153). However, they seem to respond better to immunosuppressive treatment (154, 155) and have a longer duration of articular manifestations and a lower risk of disease progression (155). In terms of natural history, RA-ILD may lead to progressive fibrosis, particularly with UIP (42, 156–159) or a widespread fibrosis (148, 160) with intercurrent acute exacerbations, with over 40% of patients fulfilling the criteria for PPF (161). It has been observed that the UIP pattern at HRCT (9, 10) is *per se* associated to an increased risk of acute exacerbation of pulmonary fibrosis, including fibrotic RA-ILD (162).

While there is no consensus or guidelines on the use and evaluation of HRCT to detect disease progression, visual evaluation is not an ideal tool to estimate the percentage of lung volume containing fibrotic features (40). Despite the absence of universal methods, the quantitative assessment (computer-based quantitative HRCT) of lung fibrosis and progression is a more objective and reproducible method (163, 164), as represented by the MeVis PULMO 3D system using the threshold value of -800HU correlating with both human observers and physiological impairment (165). A different automated quantification system includes the evaluation of lung fibrosis (as the sum of reticulation and traction bronchiectasis) and ILD (as the sum of lung fibrosis, honeycombing, and GGO) scores with a good performance in predicting prognosis in 144 patients with RA-ILD (166). Jacob et al. combined two visual staging systems in a cohort of RA-ILD patients, reaching good prognostic stratification, thus being able to identify a subpopulation of patients with progression characteristics similar to IPF (167).

### Lung ultrasonography

Lung ultrasonography (LUS) is emerging as a novel diagnostic approach for ILD (168), with the main pathologic findings being alterations in the pleural line and appearance of vertical artifacts called “B lines.” The former lesion is defined by the pleural line becoming irregular and thickened and may appear blurred and fragmented while B lines are vertical hyperechoic laser beam-like artifacts that arise from the pleural line and extend to the end of the screen without fading, erasing A lines, and moving synchronously with the pleural sliding until defining the “interstitial syndrome” (169–171). Several protocols have been proposed for LUS but there is no consensus or guidelines about the ideal examination protocol for ILD. According to different studies, LUS are able to screen for ILD in RA patients with a good sensitivity and specificity (100, 172–176) and Cogliati et al. reported that LUS is a reliable screening tool not only if performed by a trained physician using a standard 72 lines protocol but also if performed by a short-trained physician using a pocket-size lung ultrasound device (173). The presence of B lines has a sensitivity and a specificity, respectively, of 92% and 56% for RA-ILD when LUS is compared to HRCT and this is only slightly reduced (89 and 50%) when an ultrasound pocket device is used and 8 rather than 72 zones are explored (173). Results from a meta-analysis on the use of LUS diagnostic studies on RD-ILD, including RA-ILD, reaffirms the high sensitivity and specificity of LUS. Moreover, of six examined scanning protocols, a simplified method scanning only 14 lung intercostal spaces showed very high sensitivity and specificity with a short scanning time (177).

The combination of LUS with serum KL-6 demonstrated to increase the correlation with HRCT and disease severity in 150 RA cases with serum KL-6 positively correlating with LUS score and HRCT. Cut-off values of KL-6 and LUS score were 277.5 U/ml and < 5.5, with sensitivity 86.7 and 100%, and specificity 88 and 100%, respectively (100), thus confirming data from a retrospective study on patients with ILD and rheumatic diseases, including RA (178). LUS may be helpful also in the longitudinal follow-up of patients on treatment, as suggested by one case report (179).

## Lung function tests in RA-ILD

To date, there are no consensus statements nor guidelines/recommendations on functional screening and follow-up of RA-ILD. However, lung function tests are a reliable and easily accessible tool to detect lung involvement, staging disease severity, and monitor for disease progression.

Due to the systemic manifestations of RA, that can lead to musculoskeletal limitation and major exercise intolerance, lung function tests seem to be a better screening tool compared to clinical evaluation alone. Topcu et al. highlighted that symptom-related patient-reported outcome measures could be used to evaluate health-related quality of life in RA-ILD. However, they may not be very helpful in differentiating ‘ILD’ from ‘non-ILD’ causes in patients complaining respiratory symptoms, such as cough or dyspnea (180). On the other hand, concomitant comorbidities and complications due to the systemic disease involvement can represent confounding factors when assessing ILD severity (181).

RA-ILD is typically associated with a restrictive pattern with reduced carbon monoxide diffusing capacity (DLCO) on lung function tests. However, patients with RA can also develop obstructive lung disease, even in association with ILD (182).

Lung function tests can also help predicting the progression of RA-ILD. Lower forced vital capacity (FVC) and DLCO, as well as their decline over a 6-month period are associated to severe disease (159). Also, higher levels of DLCO have been associated with a better prognosis in an observational cohort (160), while DLCO ≤54% predicted has been identified as a cut-off with good sensitivity and specificity to individuate high risk of RA-ILD progression (183).

In another study enrolling 140 RA-ILD patients, most subjects experienced stable or slowly declining lung function. In 5% cases, however, rapid FVC (expressed as % predicted) deterioration was observed, especially in older adults (age > 70 years) with early diagnosis of RA. To note, the lung function trajectory did not go in parallel with RA disease activity (184).

Most RA patients are studied for lung involvement only when suggestive symptoms occur, and pulmonary disease has already evolved. However, since ILA and early ILD are present in asymptomatic patients, in our opinion it is reasonable to screen all subjects with a new diagnosis of RA with lung function tests and thoracic physical examination, looking for velcro-like crackles. Moreover, lung function monitoring and physical examination should be repeated at least once a year during follow-up; prompt radiological evaluation should be obtained in case of impaired baseline lung function tests, abnormal thoracic physical examination, or lung function decline according to recent guidelines on progressive fibrosing ILD (40).

## The current clinical practice in the management of RA-ILD

There are significant gaps in the physician knowledge regarding RA-ILD and this is well represented by the underestimated prevalence of ILD in patients with RA (185). Despite the significant burden of RA-ILD, there are no established recommendations for the management of this condition. It is disconcerting that ILD is not mentioned in the latest 2022 European Alliance of Associations for Rheumatology (EULAR) recommendations for the management of RA (186) while the 2021 American College of Rheumatology (ACR) guidelines only advise to pay attention on the role of methotrexate (MTX) in patients with a previous diagnosis of lung or airway disease without addressing ILD (187). At a local level, ILD was included in the Taiwan Society of Rheumatology recommendations for the management of comorbidities and extra-articular manifestations of RA (188), and in the Spanish Societies of Rheumatology (SER), Pneumology and Thoracic Surgery (SEPAR) guidelines for the management of RA-ILD (189, 190). In the aforementioned documents, there is general accordance against the use of MTX and leflunomide (LEF), in favor of rituximab or abatacept (188, 189), despite a recent meta-analysis found that MTX is not associated with the risk of ILD in RA (191). Remarkably, both guidelines are characterized by low quality of evidence. No recommendations or guidelines from international respiratory societies have been specifically directed towards the management of RA-ILD.

The optimal treatment choices and timing for RA-ILD have not been established and limited evidence is currently available. No RCT has investigated the role of immunosuppressants in the treatment of RA-ILD. Despite the lack of evidence, glucocorticoids are often used, and seem to be effective especially in case of NSIP and OP patterns on HRCT (192, 193). Evidence supporting the use of immunosuppressive drugs is mainly derived from large studies investigating ILD associated to systemic sclerosis (194, 195); also, in contrast to IPF, immunosuppressive therapy is safe in patients with RA-ILD also when a UIP pattern is observed (196). Cyclophosphamide (197) and mycophenolate (198) have been used with varying success, despite information on RA-ILD has been often extrapolated from studies investigating heterogeneous populations of patients with ILD associated to different rheumatic diseases (199). In a retrospective study, treatment with either azathioprine, mycophenolate or rituximab was associated with improved pulmonary function at 12 months with no difference among the treatment regimens (200), while in another retrospective study rituximab has shown some efficacy in RA-ILD patients with progressive ILD despite treatment with glucocorticoids and conventional synthetic DMARDs or immunosuppressants (201) Further evidence is required to support the use of specific immunosuppressive drugs in RA-ILD, and a precision medicine approach is warranted to target specific disease pheno- and endotypes.

Concern has been raised towards the use of anti-TNF therapy in patients with RA-ILD, since cases of disease progression and safety issues have been reported but the clinical relevance and prevalence of these observations require further data-based confirmation (202). Biologic DMARDs with targets other than TNF-alpha appear to be associated with slower rate of progression of lung disease, whereas anti-TNF therapy does not correlate with a risk of ILD worsening (203). Despite the promising role of tocilizumab, an anti-IL-6 receptor monoclonal antibody, in patients with ILD secondary to systemic sclerosis (204), further evidence is required concerning RA. Tocilizumab has demonstrated potential efficacy in maintaining lung function with a good safety profile, in a retrospective cohort of patients with RA-ILD (205). Evidence from a large retrospective registry might encourage the use of rituximab in patients with RA-ILD (206). The RECITAL trial has demonstrated efficacy and safety of rituximab in patients with connective tissue disease-associated ILD, but the study did not include RA-ILD (207). A possible role for Janus kinase (JAK)-inhibitors has been suggested from animal models of ILD associated with arthritis (208) as the JAK2 isoform specifically mediates TGF-beta signaling and the activation of myofibroblasts, and has been advocated in the molecular pathophysiology of RA-ILD (209, 210). However, due to the lack of solid evidence, the use of JAK-inhibitors cannot be encouraged for the treatment of RA-ILD (211, 212). Abatacept has shown promising results in different clinical studies (213–216); the results of the APRIL trial (NCT03084419) which is evaluating change in lung function at 24 weeks in RA-ILD patients treated with abatacept, are still expected. Iguratimod, a novel synthetic DMARD approved in Japan and China, prevents nuclear factor kappa B (NF-kB) migration into the cellular nucleus, thus impairing the transcription of proinflammatory genes and blocking the inflammatory response (217). Iguratimod has been evaluated in a study on 101 RA-ILD patients showing reduction of general inflammation, disease activity, and improvement in lung function (218).

With regard for antifibrotic molecules, these include nintedanib and pirfenidone but only the former is suggested as a therapeutic option in patients with RA ILD who meet the criteria for PPF according to ATS/ERS/JRS/ALAT Guidelines (40). In fact, the INBUILD trial demonstrated the efficacy and safety of nintedanib in patients with PPF other than IPF and significantly reduced the lung function decline at 52 weeks (219); a *post hoc* analysis found significant results in patients with autoimmune disease-related PPF without a specific analysis for RA-ILD (220).

On the other hand, pirfenidone did not achieve the same results and the TRAIL study (NCT02808871), a RCT enrolling patients with RA-ILD to compare pirfenidone to placebo, has been stopped early due to slow recruitment during the COVID-19 pandemic. However, preliminary results seem to suggest the efficacy of pirfenidone in slowing the rate of decline of FVC over time in patients with RA-ILD, although caution in interpreting results is necessary since the study was unpowered (221).

Regarding AE of RA-ILD, no consensus or management guidelines have been published, and, notably, diagnostic criteria are derived from AE in IPF (7). Few retrospective studies have analyzed AE in different RD-ILD (222) and RA-ILD alone (10, 11, 223, 224). In most cases, patients were treated with high doses steroids and best supportive care. One retrospective study failed to demonstrate benefits in term of survival from the use of cyclophosphamide in AE of RA-ILD (223). On the other hand, Ota et al. retrospectively found that the use of high doses of steroids and immunosuppressants (including cyclophosphamide, tacrolimus and cyclosporine) could improve the prognosis in AE of RA-ILD (224). Further studies are necessary to better understand pathologic mechanisms behind AEs in RA-ILD and improve management and prognosis of this severe complication.

Table 3 reports all ongoing RCTs on RA-ILD obtained from a systematic research of different registry trials (^1^ISRCTN registry and EU clinical trials register) on RA-ILD patients or trials on RA patients evaluating ILD among secondary outcomes. Unfortunately, these are based on variable encoded approaches for the diagnosis and management of RA-ILD and underlines the gaps in a uniform approach to this condition.

**Table 3:** Ongoing randomized clinical trials targeting or including RA-ILD patients.

NCT	Study type	Inclusion criteria	Intervention	Recruiting	Primary outcomes	Secondary outcomes
NCT05246293	Interventional Phase II Open Label Enrolment: 60	- ACR/EULAR 2010 RA classification criteria. - ILD (NSIP, UIP, LIP, OP) at HRCT or a surgical lung biopsy. - 18 years of age or older. - LTBI or TB excluded - Patients must discontinue using the non-permitted medications*	Tofacinib 5 mg BID for 12 months	Yes	AEs [Time frame 52 weeks]	FVC (L) DLCO (mil/min/mmHg)
						6MWT Rheumatoid arthritis disease activity according to the SDAI and DAS28
NCT04311567	Interventional Phase IV Single blind (Outcomes Assessor) Enrolment: 48	- Diagnosis of RA according to the ACR/EULAR 2010 criteria within 24 months. - No previous treatment with DMARDs. History of PDN use is allowed but should have been discontinued 2 weeks before baseline measurement. - Active disease with ≥2 painful and ≥ 2 swollen joints in 66/68 joints and CRP ≥2.0 mg/l - Aged 18–80 years	Tofacinib 5 mg BID for 48 weeks vs. Methotrexate 20 mg once weekly for 48 weeks	Yes	Total IDL score of pulmonary abnormalities by HRCT [Time frame 24 weeks]	Extent of ILD pattern by HRCT FVC DLCO 6MWD SpO2 after 6MWT Patient reported outcome of breathing and airway symptoms DAS28-CRP HAQ index DAS remission AEs Patient reported global disease activity Proportion of patients in RA ACR-EULAR Boolean remission CDAI
NCT03084419	Interventional Phase II Open Label Enrolment: 30	- Aged 18 years or over - Diagnosis of RA by 2010 EULAR/ACR criteria - RA-ILD - PF over 14 months**	Abatacept infusions 10 mg/kg fortnightly for the first 4 weeks, then every 4 weeks for a total of 20 weeks	No	FVC [Time frame 28 weeks]	DLCO mMRC K-BILD Semi-quantitative radiological scoring of the ILD SpO2 DAS28 LCQ EQ-5D Respiratory tract infection
NCT03798028	Interventional Single blind (Participant)Enrolment: 250	- 2010 ACR/EULAR classification criteria or the 1987 ACR classification criteria. - Age 18 to 70 years old.- DAS 28 ≥ 3.2 - SDAI>11.0 - CDAI >10.0 - HGB < 90 g/l and/or ILD at HRCT. - Poor response to current treatment.***	UC-MSCs intravenous injection at the dose of 1 × 10^6 cells/kg (single dose)vs.Placebo	No	Blood routine HGB FVC and/or DLCO [Time frame 24 weeks]	Remission rates of ACR 20–50-70 WBC and PLT count FVC DLCO HRCT 6MWD
NCT04928586	Interventional Phase 4 Open Label Enrolment: 200	Aged 18–80 years. In accordance with the diagnostic criteria of CTD-ILD****	DMARDs + Pirfenidone up tothe maximum tolerable dose vs. DMARDs	Yes	FVC DLCO [Time frame 12 months]	FVC, DLCO, 6MWD Dyspnea score HRCT CRP, ESR VAS score AEs
NCT05505409	Interventional Phase IV Open LabelEnrolment: 120	- Age ≥ 18 years - CTD diagnostic criteria (RA, IIM, SSc) and UCTD/IPAF classification criteria. - HRCT diagnosis confirmed ILD with corresponding clinical manifestations. - Nonresponding or progressive ILD § - Stable dose of concomitant therapy for at least 4 weeks before the baseline period.	Pirfenidone up to the maximum tolerable dose + glucocorticoid + immunosuppressantvs. Glucocorticoid + immunosuppressant	Yes	FVC [Time frame 6 months]	FEV1%, DLCO%, TLC% PFS 6MWD SpO2 HRCT SGRQ mMRC dyspnea score Clinical deterioration CRP, ERS Inflammatory factors and indicators Primary disease activity AE and SAE FVC% area under the curve Predicators of pirfenidone response in each disease subgroup
NCT00578565	Interventional Phase III Triple blind Enrolment: 123	- Diagnosis of RA according to the revised 1987 American Rheumatism Association criteria - PF (UIP or NSIP subtype) §§ - No change of DMARD treatment within the last 3 months	Rituximab 1,000 mg infusion on each day 1 and 15 with repeat dosing at 6 months.	No	DLCO FVC [Time frame 48 weeks]	Lung Fibrosis Score at HRCT DAS28 Health Associated Quality of Life
NCT02990286	Interventional Phase III Quadruple blind Enrolment: 122	- Age ≥ 18 years - A diagnosis of ILD and NSIP - Patients who did not respond or relapsed or were not able to continue at least one first-line immunosuppressive treatment of ILD ##	Rituximab 1,000 mg infusion (day 1), and 1,000 mg (day 15) + MMF 2 grams daily for 6 monthsvs. Placebo infusion (day 1 and 15) + MMF 2 grams daily for 6 months	No	FVC% [Time frame 6 months]	PFS Quality of life VAS Cough FVC, DLCO, 6MWT Cumulative doses of corticoids Autoantibodies concentration Biological markers related to lymphocyte B depletion HRCT AE

*Leflunomide, azathioprine, cyclosporine, tacrolimus, cyclophosphamide, and any biologic disease-modifying drug (bDMDARDs) such as anti-TNF therapy, rituximab, tocilizumab, etc. Patients must have a stable prednisone dose of ≤10 mg/PO/day for at least three months. All patients must have stable doses of prednisone during the last three months of follow-up, and the prednisone dose must be ≤10 mg/day. Patients without a prednisone history in the previous three months may also be included in the protocol.

^**^Progression will be defined as EITHER: a decrease in FVC by at least 5% when comparing two sets of PFTs done in the last 24 months, but with an interval of up to 14 months between the PFTs OR progression of lung fibrosis on a high-resolution CT chest, as reported by a chest radiologist.

***The current treatment refers to receive the medicines (including Leflunomide, Methotrexate, Sulfasalazine, Hydroxychloroquine, Cyclosporine A, and Tacrolimus, alone or in combination for 3 months, and maintain the stable dose of drugs for at least 1 month). More than 3 months and a stable dose for at least 1 month are required if glucocorticoid is used. The dose of glucocorticoid is less than or equal to10mg/day of prednisone.

****The diagnosis of CTD is in line with the international classification standard of rheumatism (including inflammatory myopathy, systemic sclerosis, rheumatoid arthritis, Sjogren’s syndrome, systemic lupus erythematosus, mixed connective tissue disease, undifferentiated connective tissue disease).

**§** Patients with clinical deterioration more than 1 month after diagnosis of ILD history, or poor response or intolerance to glucocorticoids or immunosuppressants treatment, or poor response or intolerance to other antifibrotic drugs (acetyl hemitrine, nidanib, etc.), or effective use of pirfenidone, and exacerbation of clinical symptoms or ILD indicators more than 3 months after withdrawal of the drug. Poor response was defined as no improvement in one of the following:

(1) Symptoms of dyspnea such as cough, chest tightness, breathlessness, shortness of breath after activity, or decreased activity endurance.

(2) the worst decrease in oxygen saturation as measured by SpO2 observed during 6MWD.

(3) There was no improvement in pulmonary ventilation (FVC%) or lung dispersion (DLCO%).

(4) HRCT findings: new onset, fibrosis tendency or density of ILD lesions were not decreased.

Clinical deterioration was defined as meeting one of three criteria:

Clinical deterioration or dyspnea within 4 weeks.

New or worsening radiological abnormalities on chest X-ray or HRCT.

Objective deterioration of pulmonary function tests or gas exchange, defined as meeting at least one of the following criteria:

(1) Start long-term oxygen therapy or increase oxygen supplementation by at least 1 l/min to maintain resting oxygen saturation of at least 90%.

(2) FVC decreased by more than 5% compared with the previously measured value; Or a decrease in DLCO of more than 10% from previous measurements; Or a 20% decrease in 6MWD from previous measurements.

§§ Clinical symptoms consistent with ILD with onset between 3 months and 36 months prior to screening.

Worsening as demonstrated by any one of the following within the past year: (1) > 10% decrease in FVC; (2) increasing infiltrates on chest X-ray or HRCT, or worsening dyspnea at rest or on exertion.

Diagnosis of UIP or NSIP by either of the following: (1) Open or VATS lung biopsy showing definite or probable UIP or NSIP; (2) HRCT scan showing definite or probable UIP or NSIP AND abnormal pulmonary function tests (reduced FVC or decreased DLCO or impaired gas exchange at rest or with exercise) AND insidious onset of otherwise unexplained dyspnea or exertion and bibasilar, inspiratory crackles on auscultation.

FVC > 50% of predicted value at Screening.

DLCO >30% of predicted value at Screening.

# A diagnosis of ILD (1) ILD associated with differentiated CTD or IPAF (based on internationally accepted criteria) (2) OR idiopathic ILD.

A diagnosis of NSIP based on: (1) a histological pattern of NSIP (2) OR HRCT findings suggestive of NSIP defined as basal predominant reticular abnormalities with traction bronchiectasis, peri-bronchovascular extension and subpleural sparing, frequently associated with ground-glass attenuation.

##: corticosteroids, azathioprine, cyclophosphamide or other immunosuppressants. For the assessment of clinical response, the absence of response was defined as: either a decrease or an increase, but <10% in % predicted FVC.

ACR/EULAR, American College of Rheumatology / European League Against Rheumatism; RA, rheumatoid arthritis; ILD, interstitial lung disease; NSIP, nonspecific interstitial pneumonia; UIP, usual interstitial pneumonia; LIP, lymphocytic interstitial pneumonia; OP, organizing pneumonia; HRCT, high resolution computed tomography; LTBI, latent tuberculosis infection; TB, tuberculosis (active disease); AE, adverse event; SAE, severe adverse event; FVC, forced vital capacity; 6MWT, 6 minute walking test; SDAI, simplified disease activity index; DAS28, Disease Activity Score Index; DMARDs, disease modifying anti-rheumatic drugs; PDN, prednisone; CRP C, reactive protein; BID, bis in die; SpO2, blood oxygen saturation; 6MWD, 6 minute walking distance; DLCO, diffusion capacity for carbon monoxide; HAQ, index health assessment of physical function index; PF, progressive fibrosis; mMRC, modified Medical Research Council dyspnea scale; K-BILD, Kings Brief Interstitial Lung Disease score; LCQ, Leicester Cough Questionnaire score; EQ-5D, Euro Quality of life 5 dimension; CDAI, Clinical Disease Activity Index; HGB, Hemoglobin; UC-MSCs, human umbilical cord blood mesenchymal stem cells; ACR 20–50-70, American College of Rheumatology 20–50-70; WBC, White blood cell; PLT, platelet; CTD-ILD, connective tissue disease interstitial lung disease; ERS, Erythrocyte Sedimentation Rate; VAS, visual analogic scales; IIM, idiopathic inflammatory myositis; SSc, systemic sclerosis; UCTD, Undifferentiated Connective Tissue Disease; IPAF, interstitial pneumonia with autoimmune features; SGRQ, St. George’s Respiratory Questionnaire; FEV1, forced Expiratory Volume in the 1st second; VATS, video-assisted thoracic surgery; MMF, Mycophenolate Mofetil; PFS, progression free survival,

The management of RA-ILD patients should be not only based on the treatment of ILD itself, but should also address the clinical consequences and comorbidities of RA. With regard to obstructive lung disease, there are no specific guidelines targeting the management in RA patients, and the impact still remains largely unexplored (46). However, smoking cessation programs should be proposed to all patients to reduce risk of death and improve quality of life (225). Patients should be screened for obstructive lung disease and treated accordingly to the current guidelines. The use of conventional DMARDs has been explored both in asthma and in COPD cohorts, and methotrexate may exert a modest steroid-sparing effect (46). As both bronchiectasis (46) and ILD (51, 52) can be associated with an increased risk of lower tract respiratory infections, a multidisciplinary approach including pulmonologists and rheumatologists is warranted for all patients, in order to evaluate the best pharmacologic interventions and reduce the risk of infections (46, 51, 52). Moreover, microbiological sampling should be considered in case of infection, particularly pneumonia, and DMARDs should be suspended and recommenced only once the antibiotic therapy is completed and clinical symptoms have resolved (226). Pneumococcal and annual Influenza vaccinations should be offered to all patients with RA, regardless of the treatment (226, 227), along with SARS-CoV-2 immunization (228, 229). Finally, since treatment with anti-TNF therapy is associated with an increased risk of developing TB, screening and treatment for latent TB should be proposed to all RA patients (226).

Pulmonary hypertension secondary to ILD has been associated to reduced exercise capacity, increased need for supplemental oxygen, worse quality of life and prognosis (230–232). Screening for pulmonary hypertension should be performed in all RA-ILD patients although no recommendation on timing and frequency is available (233). Recently, the INCREASE trial (234) has shown significant improvements in exercise capacity in ILD patients with PH treated with inhaled treprostinil. Clinical worsening also occurred less frequently in the treprostinil group, compared with placebo. The trial also included RD-ILD patients, but subgroup analysis has not been performed, and targeted clinical trials are warranted to confirm these results in specific populations, as is the case of RA-ILD patients. Since subjects with RA-ILD are at higher risk of developing malignancy and in particular lung neoplasms (22, 24), cancer screening should be systematically performed; however, there is no clear indication regarding timing and frequency (24).

In addition to clinical comorbidities, several relevant treatable traits have been identified in ILD and should be addressed in RA-ILD (235), including dyspnea, exercise-induced hypoxemia, and exercise intolerance. In RA-ILD patients, these conditions can be worsened by musculoskeletal involvement due to RA itself. Referral to pulmonary rehabilitation should be considered as an important component of comprehensive patient care (236). On the other hand, in case of end stage disease, a palliative approach is preferable to reduce the burden of symptoms and improve the quality of life (237). Finally, lung transplantation could be considered in selected patients with RA-ILD; no significant differences have been described in terms of survival, acute and chronic rejection, or extrapulmonary organ dysfunction compared to IPF (238, 239). Thus, lung transplant could offer a chance to improve the quality of life in the appropriate patients’ subsets (239).

## Conclusion

Available data on RA-ILD epidemiology remain unconclusive and heterogeneous for both clinical and research purposes and significant more research efforts are required to finely define incidence, prevalence, and mortality of ILD in the RA population. In particular, one priority is the harmonization of the detection methods since HRCT is the gold standard technique for the diagnosis of ILD. Second, it is essential to define which patients with RA are at increased risk of ILD and, thus, deserve early radiologic investigations as delayed diagnosis is associated with increased mortalityss. Third, the timing, frequency, and the potential role of alternative screening methods, such as lung function tests, serum biomarkers and LUS, also need to be determined (26), likely with the use of biomarkers, including both autoantibodies and non-autoimmune biomarkers. Fourth, the proportion of patients with radiologic ILD who will progress to clinically overt disease is unknown, as is the proportion of patients with radiologic ILD who might benefit from early treatment. Ultimately, efforts are required to imbricate clinical, biological, radiological, and functional risk factors to find reproducible prediction models to estimate the risk and prognosis of ILD in the RA population and to stratify patients with RA-ILD at risk of developing PPF.

## Author contributions

AS, AT, and GB: review of relevant papers and manuscript preparation. All authors contributed to the article and approved the submitted version.

## Conflict of interest

The authors declare that the research was conducted in the absence of any commercial or financial relationships that could be construed as a potential conflict of interest.

## Publisher’s note

All claims expressed in this article are solely those of the authors and do not necessarily represent those of their affiliated organizations, or those of the publisher, the editors and the reviewers. Any product that may be evaluated in this article, or claim that may be made by its manufacturer, is not guaranteed or endorsed by the publisher.

## Footnotes

^1^ Clinicaltrial.gov
